# Promoting the process of determining brain death through standardized training

**DOI:** 10.3389/fneur.2024.1294601

**Published:** 2024-02-22

**Authors:** Yingying Su, Yan Zhang, Hong Ye, Weibi Chen, Linlin Fan, Gang Liu, Huijin Huang, Daiquan Gao, Yunzhou Zhang

**Affiliations:** ^1^Brain Injury Evaluation Quality Control Center of the National Health Commission, Beijing, China; ^2^Xuanwu Hospital Capital Medical University, Beijing, China

**Keywords:** clinical investigation, practical guidance, training model, brain death determination, standard, practical, training mode

## Abstract

**Objective:**

This study aims to explore the training mode for brain death determination to ensure the quality of subsequent brain death determination.

**Methods:**

A four-skill and four-step (FFT) training model was adopted, which included a clinical neurological examination, an electroencephalogram (EEG) examination, a short-latency somatosensory evoked potential (SLSEP) examination, and a transcranial Doppler (TCD) examination. Each skill is divided into four steps: multimedia theory teaching, bedside demonstration, one-on-one real or dummy simulation training, and assessment. The authors analyzed the training results of 1,577 professional and technical personnel who participated in the FFT training model from 2013 to 2020 (25 sessions), including error rate analysis of the written examination, knowledge gap analysis, and influencing factors analysis.

**Results:**

The total error rates for all four written examination topics were < 5%, at 4.13% for SLSEP, 4.11% for EEG, 3.71% for TCD, and 3.65% for clinical evaluation. The knowledge gap analysis of the four-skill test papers suggested that the trainees had different knowledge gaps. Based on the univariate analysis and the multiple linear regression analysis, among the six factors, specialty categories, professional and technical titles, and hospital level were the independent influencing factors of answer errors (*p* < 0.01).

**Conclusion:**

The FFT model is suitable for brain death (BD) determination training in China; however, the authors should pay attention to the professional characteristics of participants, strengthen the knowledge gap training, and strive to narrow the difference in training quality.

## Introduction

In 2020, “Determination of Brain Death/Death by Neurological Criteria: The World Brain Death Project,” which emphasized that “the determination of brain death (BD) should be completed by licensed doctors who have received training and independent medical qualification” was published (1). In China, although two versions of BD determination criteria and practical guidance have been implemented/introduced (2–4), the strategies and methods to carry out standardized training, to enable more professional technicians to master BD determination technology, and to advance the process of BD determination in a shorter time have become new challenges and topics. It is related to the quality of brain death determination and subsequent medical decisions. This study analyses the training results of professional and technical personnel (1,577 people, 2,355 person-times) who received standardized training in China from 2013 to 2020 to explore whether the training mode is suitable for China’s national conditions and whether the training quality can be improved through improvement and perfection, by improving the training content that has not been covered or addressed or refining the training focus for different participants. Perhaps, the relevant opinions or suggestions may have certain reference significance for countries or regions that have not yet carried out but are ready to carry out standardized training on BD.

## Materials and methods

### Training materials

From 2013 to 2020, the Brain Injury Evaluation Quality Control Center (BQCC) held 25 sessions of standardized training on the “Criteria and practical guidance for determination of brain death.” All participants were qualified licensed doctors or technicians and had at least 5 years of clinical work experience or 2 years of skill operation experience. The trainees can register for one or more training programs independently.

### Training methods

(1) The standardized training mode for the determination of brain death (BD) (Table 1) was adopted for all personnel in the whole training process, which mainly included four skills and was divided into four steps (four skills and four steps of training, FFT). The whole process was completed offline in the teaching hospital by the BQCC teacher team (professional and technical personnel with standardized training of teachers).

**Table 1:** Standardized training mode of brain death determination.

	Step one Theoretical training		Step two Demonstration training	Step three Simulation training	Step four Examination^b^ and explanation^c^
Training content^a^	Definition				
	Preconditions				
	Evaluation criteria				1 essay question
	Evaluation process				
	Clinical evaluation	Deep coma. Absence of brainstem reflex. Absence of spontaneous breathing. AT.	Deep coma. Absence of brainstem reflex. Absence of spontaneous breathing. AT.	Deep coma. Absence of brainstem reflex. Absence of spontaneous breathing. AT.	40 multiple-choice questions 13 knowledge points
	Confirmatory tests	EEG	EEG	EEG	20 multiple-choice questions 5 knowledge points
		SLSEP	SLSEP	SLSEP	20 multiple-choice questions 6 knowledge points
		TCD	TCD	TCD	20 multiple-choice questions 6 knowledge points
Training methods	Multimedia teaching for all trainees		Bedside item teaching	Simulator exercise	Itemized examined
Training duration	4 class hours		1 class hour for each item	1 class hour for each item	45 min per item

^a^The four operation specifications include clinical examination, EEG, SLSEP, and TCD; ^b^Each examination includes 1 essay question and 20 or 40 multiple-choice questions; ^c^After the end of the examination, analyze and explain the test questions, that is, the last intensive training; AT, apnea test; EEG, electroencephalogram; SLSEP, short-latency somatosensory evoked potential; TCD, transcranial Doppler.

1. Theoretical training: Multimedia teaching and discussion enabled participants to fully understand the historical background, purpose, significance, and theoretical basis of BD determination. The core content of the theoretical training included the definition of BD, prerequisites, criteria of the determination, evaluation process, operational specifications of clinical evaluation (deep coma, absence of brainstem reflexes, no spontaneous respiration, and apnea test), confirmatory tests (an electroencephalography (EEG), a short-latency somatosensory evoked potential (SLSEP), and a transcranial Doppler (TCD)), and operational specifications (environmental requirements, equipment requirements, parameter setting, operational methods and steps, inspection location, recording specifications, interpretation of results, influencing factors, and precautions). The training time was four class hours.
2. Demonstration training: The demonstration training was divided into four steps: a clinical evaluation, an EEG evaluation, an SLSEP evaluation, and a TCD evaluation. The instructors demonstrated the operation skills at the bedside of the neurointensive care unit (neuro-ICU) or intensive care unit (ICU), focusing on the problems that may be encountered in the operational process and solutions. Participants could experience the bedside implementation process of various skills through observation (one class hour for each skill and four class hours in total).
3. Simulation training: The simulation training was divided into four steps: a clinical evaluation, an EEG evaluation, an SLSEP evaluation, and a TCD evaluation. In the demonstration room, one-to-one practice session with either a dummy or a real person was conducted. That is, after the teacher demonstrated the operation process, the trainee staff learned and practiced with each other under the guidance of the instructor, thereby mastering standardized operation skills (four class hours for each skill).
4. Examination and analysis: All trainee staff received an analysis of their written examination (the last intensive training) so that they could understand the reasons for their incorrect answers and correct them to eliminate doubts and blind spots (two class hours).

(2) Examination methods

The examination was divided into two parts: simulation investigation (in the process of simulation training) and written test completion. The content of the written test was designed according to the “Criteria and practical guidance for the determination of brain death” published in China (2–4) and included four types of questions, namely, clinical evaluation (13 knowledge points; 1 essay question and 40 multiple-choice questions), EEG determination (5 knowledge points; 1 essay question and 20 multiple-choice questions), SLSEP determination (6 knowledge points; 1 essay question and 20 multiple-choice questions), and TCD determination (6 knowledge points; 1 essay question and 20 multiple-choice questions). The answer time for each test was 45 min.

### Statistical analysis

In total, 2,355 participant tests were analyzed in SPSS 17.0 statistical software. SPSS statistical software, version 22.0 (SPSS Institute, Inc., Chicago, IL, USA), was used for all statistical analyses. The authors calculated the error rate for each knowledge point (error rate = number of wrong answers to a knowledge item/total number of knowledge items) and total knowledge (total number of wrong answers per test). For example, there were 4 questions evaluating the pupillary light reflex, and 40 people answered every question. The numbers of incorrect responses to each question were 2, 3, 4, and 5. Thus, the number of incorrect responses to this knowledge point = 2 + 3 + 4 + 5; total number of questions = 4 * 40; error rate = 14/160 * 100%, and error rate (%) = 90/1766 * 100% = 5.1%. The authors performed univariate comparisons of the error rate with Fisher’s exact tests. Multiple linear regression was used for multivariate analysis. The answer results (number of errors) were the independent variables, while age, gender, specialty categories, professional and technical titles, and hospital level were the dependent variables. The independent influencing factors of the answer results were analyzed. *p* < 0.05 was considered statistically significant.

## Results

1. Training sessions were conducted from 2013 to 2020. The trainees came from 379 hospitals, covering 31 provinces on the Chinese mainland. There were a total of 1,577 trainees and 2,355 training person-times, including 1,179 person-times for clinical diagnosis training, 454 person-times for EEG training, 345 person-times for SLSEP training, and 377 person-times for TCD training (Table 2).
2. Error rate analysis of the written examination.

**Table 2:** Basic information of trainers.

	Clinical testing *N* = 1,179	EEG testing *N* = 454	SLSEP testing *N* = 345	TCD testing *N* = 377
Age (year)				
25–29	67 (5.7%)	94 (20.7%)	83 (24.1%)	79 (21.0%)
30–39	553 (46.9%)	227 (50.0%)	167 (48.4%)	190 (50.4%)
40–49	450 (38.2%)	111 (24.4%)	72 (20.9%)	89 (23.6%)
50–59	109 (9.2%)	22 (4.8%)	23 (6.7%)	19 (5.0%)
Sex				
Male	730 (61.9%)	189 (41.6%)	134 (38.8%)	158 (41.9%)
Female	449 (38.1%)	265 (58.4%)	211 (61.2%)	219 (58.1%)
Specialty categories				
Neurology	505 (42.8%)	225 (49.6%)	177 (51.3%)	180 (47.7%)
Neurosurgery	229 (19.4%)	46 (10.1%)	40 (11.6%)	40 (10.6%)
ICU or emergency	359 (30.4%)	90 (19.8%)	64 (18.6%)	78 (20.7%)
Other	86 (7.3%)	93 (20.5%)	64 (18.6%)	79 (21.0%)
Professional categories				
Physician	1,177 (99.8%)	394 (86.8%)	294 (85.2%)	325 (86.2%)
Technician	2 (0.2%)	60 (13.2%)	51 (14.8%)	52 (13.8%)
Professional and technic titles				
Junior (Resident)	92 (7.8%)	111 (24.4%)	93 (27.0%)	87 (23.1%)
Middle (Attending Physician)	415 (35.2%)	204 (44.9%)	155 (44.9%)	164 (43.5%)
Senior (Chief Physician)	672 (57%)	139 (30.6%)	97 (28.1%)	126 (33.4%)
Hospital level				
Grade 3 class A hospital	1,087 (92.2%)	431 (94.1%)	329 (95.4%)	356 (94.4%)
Other	92 (7.8%)	23 (5.1%)	16 (4.6%)	22 (5.8%)

**Figure 1:**
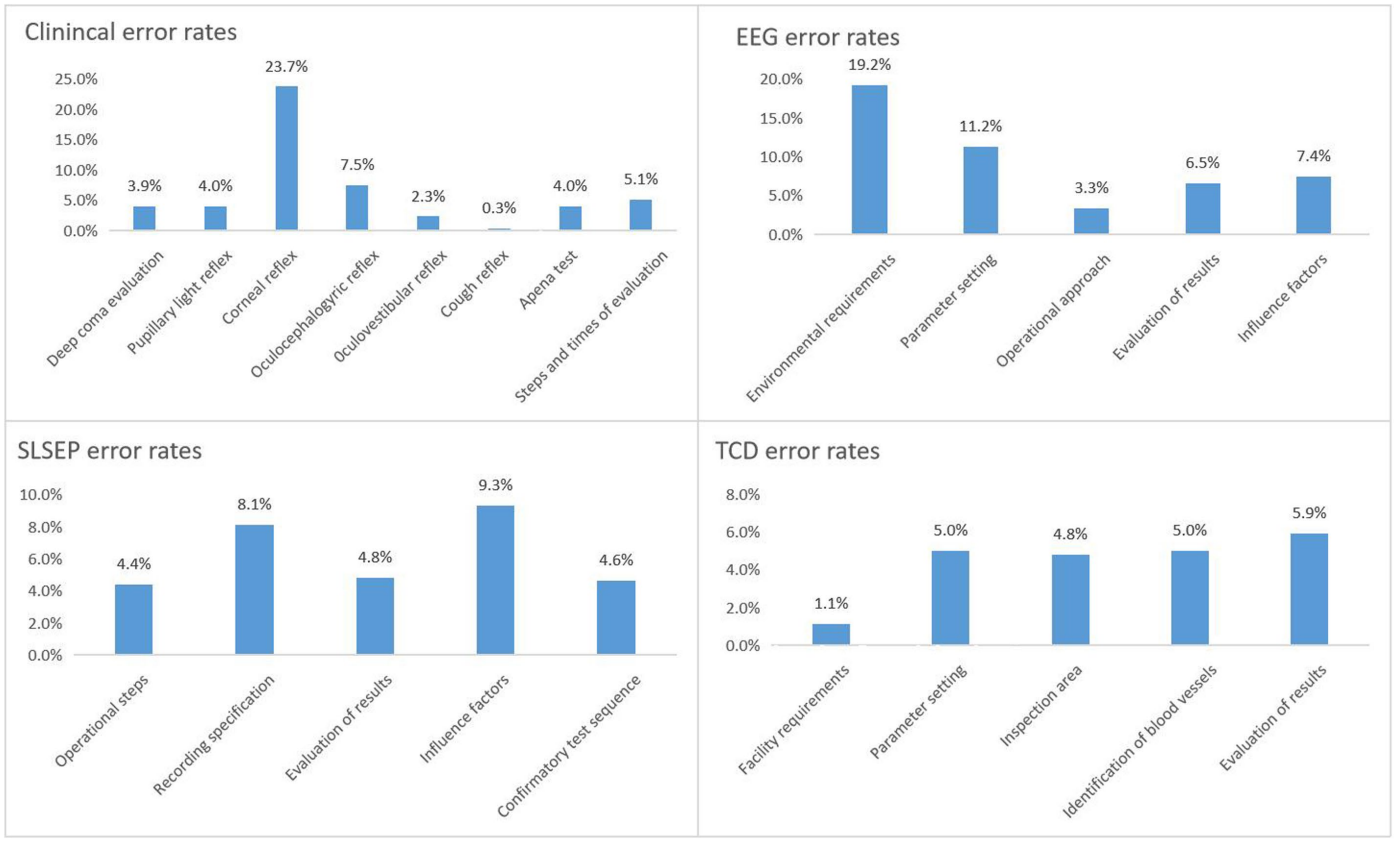
Error rate analysis of knowledge point of four techniques. EEG, electroencephalogram; SLSEP, short-latency somatosensory evoked potential; TCD, transcranial Doppler.

The total error rate of the written test was assumed to reflect the level of mastery of the published criteria and the practical guidance acquired for the determination of BD. The total error rates for all four written examination topics were < 5%, at 4.13% for SLSEP, 4.11% for EEG, 3.71% for TCD, and 3.65% for clinical evaluation. The total accuracy rates of the four confirmatory tests were 41.38% for TCD, 41.16% for SLSEP, 27.59% for EEG, and 22.56% for clinical evaluation. As shown in Figure 1, differences in the level of mastery of knowledge and skills among the four items were observed. The analysis of knowledge points more accurately revealed the knowledge gaps between the trainees and the teaching gaps of the trainers (Figure 1).

3 Analysis of factors related to errors.

**Figure 2:**
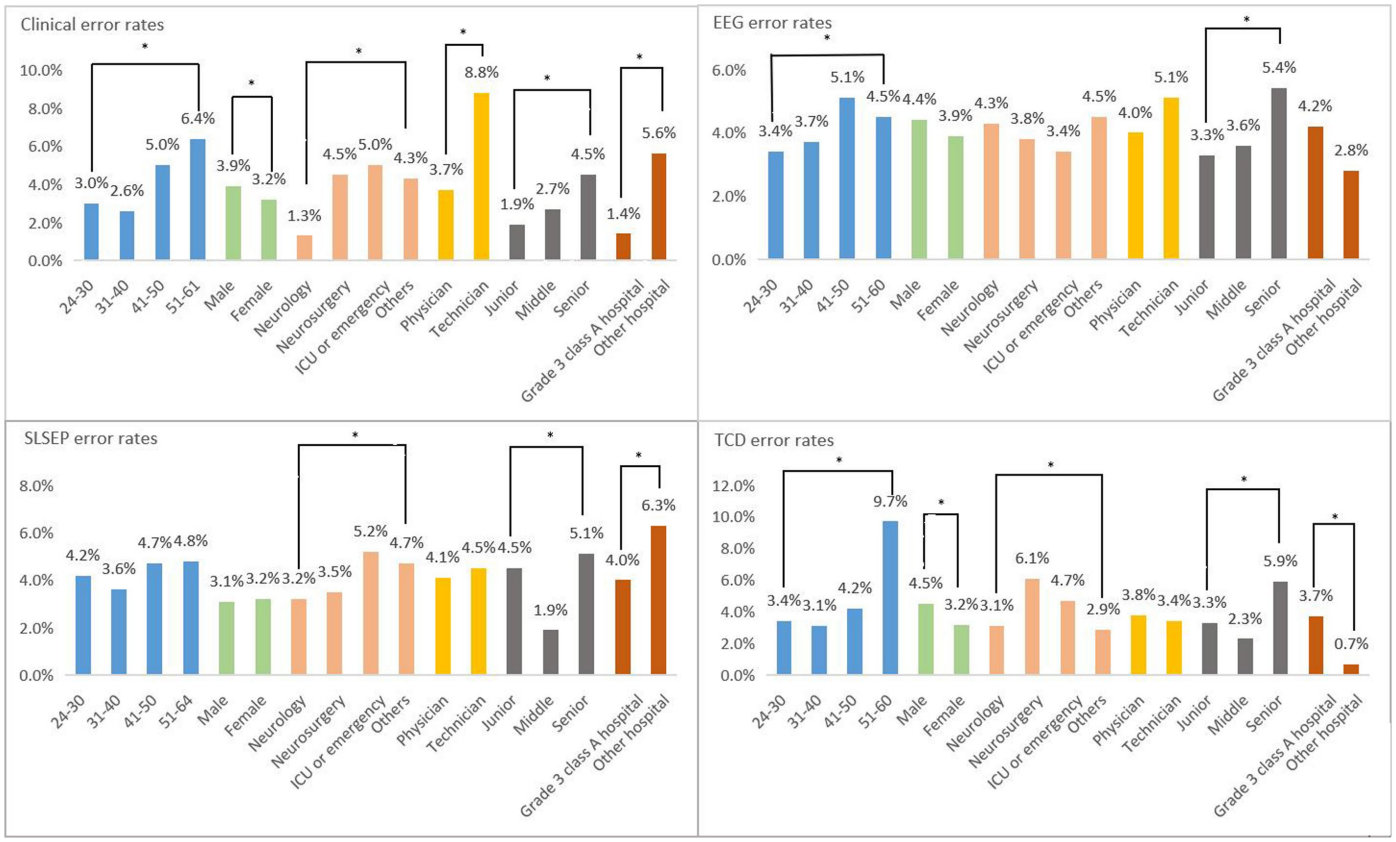
Univariate analysis of influencing factors of four techniques. Clinical: significant differences were observed in all clinical items. EEG (electroencephalogram): significant differences were observed in age and professional title. SLSEP (short-latency somatosensory evoked potential): significant differences were observed in specialty categories, professional title, and hospital level. TCD (transcranial Doppler): significant differences were observed in all except professional categories. *p < 0.05.

The influencing factors of answer errors were identified to refine the training objectives. Based on the univariate analysis (Figure 2) and the multiple linear regression analysis (Table 3), among the six factors, specialty categories, professional and technical titles, and hospital level were the independent influencing factors of clinical evaluation answer errors (*p* < 0.01). The professional and technical title was the independent influencing factor of incorrect answers of the EEG confirmatory test (*p* < 0.001). The specialty categories and professional technical titles were the independent influencing factors of the SLSEP confirmatory test answer errors. The professional and technical title was the independent influencing factor of the TCD confirmatory test answer errors.

**Table 3:** Multivariate linear regression analysis of all factors influencing error volume.

	Variables	*B*	SE	*t*	Sig.
Clinical error	Age	0.062	0.007	8.488	0.000
	Specialty categories	0.384	0.112	3.433	0.001
	Hospital level	0.597	0.210	2.842	0.005
EEG error	Professional title	0.510	0.140	3.633	0.000
SLSEP error	Specialty categories	0.499	0.178	2.806	0.005
	Professional title	0.508	0.188	2.705	0.007
TCD error	Professional title	0.659	0.160	4.125	0.000

B, coefficient; SE, standard error.

## Discussion

### Clinical evaluation training for BD determination

Clinical evaluation is the core part of the diagnosis of BD. In 1968, Harvard University proposed four criteria for BD evaluation. In addition to the electroencephalogram showing electrical silence (≤2 μv), the other three were clinical neurological examinations (5). Since then, although various countries have implemented clinical evaluation criteria for BD (2–4, 6–8), the core content has not changed significantly. The core content has played an important role in the determination of BD, so it has inevitably become the top priority of training. Our study showed that the largest number of people participated in the clinical evaluation skills training (1,179/2355, 50.06%). Although the total error rate of 13 knowledge points was only 3.65%, the accuracy rate of all answers in the test paper ranked last (22.56%). It is suggested that, even if one has 5 years of clinical experience, he or she still needs special skills training for BD evaluation. When the authors further analyzed the incorrect answers, they found that the highest error rate (23.7%) was for the evaluation of corneal reflex, while the error rates of other examination items were < 7.5%. This is consistent with the findings of Maciel et al. (9), and the misjudgment of corneal reflex examination is a concern that needs to be emphasized in training. The independent influencing factors related to clinical evaluation errors were working at non-grade 3 class A hospitals, being a non-neurology doctor, and being a doctor with a high-level professional title. An accurate understanding and judgment of a nervous system examination come from the accumulation of professional knowledge and clinical experience, especially for non-neurologists, who need to receive strict standardized training. Therefore, training staff should adjust their focus of training, especially in one-to-one simulation training, to include the cooperation of neurologists and other specialists, thus improving the quality of training.

### EEG confirmatory test training for BD determination

The application of EEG technology to confirm BD was established at the same time as the clinical evaluation of BD. Since then, this approach has become widespread. Studies have confirmed that the sensitivity and specificity of BD confirmation with whole-brain EEG showing electrical silence (≤2 μv) are 83 and 97%, respectively (the false-positive rate is only 3%) (9). However, EEG also has technical “defects,” that is, it is easily affected by anesthetic and sedative drugs, hypothermia, and metabolic disorders (10–12). These “defects” will inevitably become the key part of the EEG confirmatory test training. In this study, the number of person-times of EEG confirmatory test training ranked second (545/1179, 23.14%), the total error rate of five knowledge points (4.11%) ranked second only to SLSEP, and the accuracy rate of all answers in the test paper ranked second to last (27.59%). It is suggested that, although the EEG technology has been used in the clinic for many years, the standardization of operations is not enough, especially for the special requirements and regulations for the determination of BD with EEG. When the authors further analyzed the wrong answers, they found that the highest error rates were for EEG operating environment requirements (19.2%) and parameter settings (11.2%). The independent influencing factor related to EEG errors was being a doctor with a high-level professional title. We speculate that, in the bedside demonstration and one-to-one simulation training sessions, personnel who were trained were not familiar with the characteristics of EEG equipment and operation specifications, and we suggest that teachers should focus on this group and intensify their training. A global expert consensus on BDD, launched in 2020, no longer recommends using EEG alone to determine BD (1). However, it should be emphasized that the consensus does not negate the use of EEG in conjunction with other auxiliary tests (such as SLSEP) for BD determination in special cases. Given China’s medical and economic conditions and the advantages of EEG bedside operability, non-invasiveness, and reliability, the authors retain the EEG training program.

### An SLSEP confirmatory test training for BD determination

The application of SLSEP technology to confirm BD began in the 1980s (12). Since then, many studies have confirmed the existence of N9 and/or N13 of bilateral median nerve SLSEP and the absence of p14, N18, and N20 as the parameters of BD confirmatory tests; the sensitivity is as high as 100%, and the specificity is approximately 78–100% (the false-positive rate is approximately 0–22%) (13–16). The greatest advantage of SLSEP is that it is rarely affected by anesthetic and sedative drugs; however, it has not been popularized due to the insufficient promotion of the technology. In this study, the lowest number of people completed the SLSEP confirmatory test (345/2355, 14.64%), and although the total error rate of five knowledge points ranked first (4.13%), the correct rate of all answers in the test paper ranked second (41.16%). This finding suggested that, while most participants could master SLSEP technology well, they might need more detailed standardized training. When the authors further analyzed the incorrect answers, they found that the “record specifications” (8.1%) and “influencing factors” (9.3%) had the highest error rates. The independent influencing factors related to SLSEP errors were being a non-neurologist and having a high-level professional title. Therefore, teachers should focus their training on this group, provide them with more training opportunities, and adopt the strategy of increasing training time when necessary to allow them to truly master this skill.

### TCD ultrasound confirmatory test training for BD determination

The confirmation of BD by TCD technology is very similar to that by SLSEP technology (17). Relevant studies have confirmed that the TCD blood flow spectrum shows reverberating flow and a small systolic spike or the absence of a blood flow signal during BD (17–23), and the sensitivity and specificity for confirming BD are approximately 73–95% and 75–99% (false-positive rate is approximately 1–25%) (13, 24, 25). TCD has the advantages of non-invasiveness and strong repeatability; however, it is most vulnerable to the operator’s technical proficiency and operation experience, making training the most difficult aspect. The number of people completing the TCD confirmatory test training in this study was second only to those completing the EEG training (377/2355, 16.00%). Furthermore, the correct rate of all test questions was the highest (41.38%), and the total error rate of five knowledge points (3.71%) was also lower than that for EEG and SLSEP. This showed that TCD technology had not only a high popularity rate but also a strong subjective initiative among supervisors who mastered this skill. When the authors further analyzed the incorrect answers, they found that the error rate of each knowledge point was not high. The independent influencing factor related to TCD errors was also having high-level professional titles, which was basically consistent with the problems encountered by EEG and the training strategies that need to be adjusted.

## Conclusion

In China, EEG, SLSEP, and TCD confirmatory tests should be enforced after the clinical evaluation of BD (the minimum evaluation criteria) not only because of their bedside operability, non-invasiveness, and reliability but also because of their high popularity throughout the country (26). The authors hope that, through continuous improvement and perfection of the FFT training mode, more professional and technical personnel can carry out BD determination in a standardized manner and lay a good foundation for the subsequent construction of BD determination teams, with the hospital as the basic unit, to allow quality control of BD determination cases.

## Data availability statement

The original contributions presented in the study are included in the article/supplementary material, further inquiries can be directed to the corresponding author.

## Ethics statement

Ethical review and approval was not required for the study on human participants in accordance with the local legislation and institutional requirements. Written informed consent from the patients/participants or patients/participants’ legal guardian/next of kin was not required to participate in this study in accordance with the national legislation and the institutional requirements.

## Author contributions

YS: Conceptualization, Supervision, Validation, Writing – original draft, Writing – review & editing, Methodology. YaZ: Data curation, Supervision, Writing – review & editing. HY: Data curation, Supervision, Writing – review & editing. WC: Data curation, Supervision, Writing – review & editing. LF: Data curation, Writing – review & editing. GL: Data curation, Writing – review & editing. HH: Data curation, Writing – review & editing. DG: Data curation, Writing – review & editing. YuZ: Data curation, Writing – review & editing.
